# Labor Epidural Analgesia to Cesarean Section Anesthetic Conversion Failure: A National Survey

**DOI:** 10.1155/2019/6381792

**Published:** 2019-06-02

**Authors:** Neel Desai, Andrew Gardner, Brendan Carvalho

**Affiliations:** ^1^Department of Anaesthetics, Guy's and St Thomas' NHS Foundation Trust, Westminster Bridge Road, SE1 7EH London, UK; ^2^Department of Anesthesiology, Perioperative and Pain Medicine, Stanford University School of Medicine, Stanford, 94305 California, USA

## Abstract

**Background:**

If conversion of labor epidural analgesia to cesarean delivery anesthesia fails, the anesthesiologist can be confronted with a challenging clinical dilemma. Optimal management of a failed epidural top up continues to be debated in the absence of best practice guidelines.

**Method:**

All members of the Obstetric Anaesthetists' Association in the United Kingdom were emailed an online survey in May 2017. It obtained information on factors influencing the decision to utilize an existing labor epidural for cesarean section and, if epidural top up resulted in no objective sensory block, bilateral T10 sensory block, or unilateral T6 sensory block, factors influencing the management and selection of anesthetic technique. Differences in management options between respondents were compared using the chi-squared test.

**Results:**

We received 710 survey questionnaires with an overall response rate of 41%. Most respondents (89%) would consider topping up an existing labor epidural for a category-one cesarean section. In evaluating whether or not to top up an existing labor epidural, the factors influencing decision-making were how effective the epidural had been for labor pain (99%), category of cesarean section (73%), and dermatomal level of blockade (61%). In the setting of a failed epidural top up, the most influential factors determining further anesthetic management were the category of cesarean section (92%), dermatomal level of blockade (78%), and the assessment of maternal airway. Spinal anesthesia was commonly preferred if an epidural top up resulted in no objective sensory block (74%), bilateral T10 sensory block (57%), or unilateral T6 sensory block (45%). If the sensory block level was higher or unilateral, then a lower dose of intrathecal local anesthetic was selected and alternative options such as combined-spinal epidural and general anesthesia were increasingly favored.

**Discussion:**

Our survey revealed variations in the clinical management of a failed epidural top up for cesarean delivery, suggesting guidelines to aid decision-making are needed.

## 1. Introduction

Between 2017 and 2018, over 100 000 emergency cesarean deliveries were carried out in England, 21% of which were undertaken with epidural anesthesia alone [1]. If a cesarean delivery is needed in a parturient with an existing labor epidural, it is common practice to convert or “top up” the epidural catheter [2], with the aim of initiating surgical anesthesia by injecting more concentrated local anesthetic, usually combined with a lipid soluble opioid. Successful neuraxial anesthesia conversion is a useful measure of quality and safety, indicating the prior presence of functional labor epidural analgesia and limiting the use of general anesthesia in obstetrics [3].

Labor epidural top up for cesarean section can fail, the incidence of which has been reported to range from 0% to 21% [4]. If conversion of labor epidural analgesia to cesarean delivery anesthesia fails, the anesthesiologist can be confronted with a challenging clinical dilemma. Optimal management of a failed epidural top up is controversial in the absence of best practice guidelines and subsequent anesthetic options include the following: manipulation or replacement of the epidural; performance of a combined spinal-epidural (CSE) or spinal; and induction of general anesthesia [5]. In an effort to raise the clinical standard, guidelines from the Royal College of Anaesthetists state that the rate of conversion from neuraxial to general anesthesia should be less than 15% and 5%, respectively, for category-one cesarean section and for category-one to -three cesarean section overall [6].

In view of the lack of consensus and guidance for what constitutes the most frequently or optimal considered practice in the setting of a failed labor epidural top up for cesarean delivery, we conducted a survey to assess current practice. Our aim was to determine, in the setting of an emergency cesarean section, the decision-making process underlying whether or not anesthesiologists with an obstetric interest would top up an existing labor epidural and how the clinical dilemma of an epidural top up failure would be addressed. Further, we sought to add to the evidence relating to complications following neuraxial techniques in the situation of a failed epidural top up.

## 2. Materials and Methods

Our survey was created by two anesthesiologists with expertise in obstetric anesthesia and consisted of a maximum of 14 questions. It was considered by the Obstetric Anaesthetists' Association (OAA) survey subcommittee and tested for comprehension and logic with a cohort of 10 anesthesiologists. Comments and suggestions received were reviewed and the final survey asked about the following: grade of the respondent; standard anesthetic technique for an elective cesarean section; if an existing labor epidural would be considered for top up in the context of a category-one cesarean section; and factors that would influence decision-making when evaluating as to whether or not to top up an existing labor epidural for a cesarean section. It then enquired as to the respondents' usual next management step, be it CSE, general anesthesia, repeat epidural, spinal, or withdrawing the epidural catheter already in situ, in response to scenarios where a top up of an existing labor epidural for a category-two cesarean section resulted in one of three outcomes: no objective sensory block; good bilateral T10 dermatomal level of sensory block to temperature change; or good unilateral T6 dermatomal level of sensory block to temperature change. Respondents were told to assume that neither further epidural top ups nor time would result in any change in the dermatomal level of the sensory block, and assessment of the parturient would demonstrate no undue concerns about the airway and no obvious difficulties in achieving a neuraxial technique if needed. If, after a failed or inadequate epidural top up, in any one of these scenarios, the respondents chose to perform a CSE or spinal, we asked what dose of intrathecal local anesthetic, compared to their routine clinical practice, they would use. Our survey lastly enquired about complications which had previously been encountered by respondents in the setting of a failed epidural top up for a cesarean section and included high or total spinal consequent to CSE or spinal, inadequate sensory block needing general anesthesia following a reduced spinal dose, and local anesthetic toxicity secondary to a de novo epidural top up.

Subsequent to approval of the survey by the OAA, all OAA members in the United Kingdom were emailed to invite them to complete the online survey on 16th May 2017, and, after two emailed reminders, it was closed on 16th August 2017.

### 2.1. Statistical Analysis

Data from returned surveys were exported into a Microsoft Excel spreadsheet and information from incomplete questionnaires was not excluded. Descriptive statistics was used to summarize practice data and all results have been expressed as number (percentage). Data analysis was carried out using statistical software SPSS (Version 23, IBM Corp, Troy, NY, USA). Differences in responses between consultants and trainees were compared using the chi-squared test. In the various scenarios, differences in the usual next management step and the dose of intrathecal local anesthetic, when CSE or spinal was selected, were compared using the chi-squared test. A *p* value less than 0.05 was considered statistically significant for these comparisons.

## 3. Results

Out of the 1742 online surveys which were sent, we received 710 responses with an overall response rate of 40.8%. Five-hundred and sixty-three (79.3%) were completed by consultants, 10 (1.4%) by associate specialists, 31 (4.4%) by staff grades, and 106 (14.9%) by trainees.

### 3.1. Standard Anesthetic Technique for an Elective Cesarean Section

For an elective cesarean section, the chosen standard anesthetic technique did not differ according to the anesthesiologist's level of experience and the most common procedure was a spinal, used by 634 (90.4%) respondents. Sixty-seven (9.6%) respondents performed a CSE and no respondents used an epidural or general anesthesia as their standard anesthetic technique.

### 3.2. Use of an Existing Labor Epidural for a Cesarean Section

In evaluating whether or not to top up an existing labor epidural for a cesarean section, the most influential factor, reported by 701 (98.7%) respondents, was how effective the epidural had functioned for the management of labor pain (Table 1). Five-hundred and nineteen (73.1%) respondents were influenced by the classification of the urgency of cesarean section in making this decision. Compared to consultants, trainees were more likely to be influenced by the assessment of the maternal airway (34.8% vs 49.1%, *p*=0.005), fasting status (11.2% vs 20.8%, *p*=0.007), and the length of time the epidural had been in situ (12.1% vs 28.3%, *p* < 0.001). Six-hundred and thirty-four (89.3%) respondents would consider topping up an existing labor epidural for a category-one cesarean section and their level of expertise did not have any relationship with this decision.

### 3.3. Management of a Failed Epidural Top up of an Existing Labor Epidural for a Cesarean Section

In the setting of a failed epidural top up of an existing labor epidural for a cesarean section, the most influential decision-making factors were the category of cesarean section (91.5%), assessment of the maternal airway (77.6%), dermatomal level of the block (78.0%), perceived risk of high or total spinal (72.3%), and the pattern of neuraxial block failure such as unequal or unilateral block (68.2%) (Table 2). Relative to consultants, trainees were less likely to be influenced by the potential if needed for extension of sensory block with a CSE or epidural technique (29.7% vs 19.8%, *p*=0.04).

If a top up of an existing labor epidural resulted in no objective sensory block, bilateral T10 dermatomal level of sensory block or unilateral T6 dermatomal level of sensory block, 524 (73.9%), 398 (56.9%), and 310 (44.9%) respondents, respectively, would perform a spinal as their usual next management step (Table 3). No significant differences between consultants and trainees were found in this regard, but trainees were more likely to perform general anesthesia after bilateral T10 dermatomal level of sensory block (23.6% vs 15.6%, *p*=0.045).

For those who selected to perform a CSE or spinal as their usual next management step in response to no-objective sensory blockade, bilateral T10 dermatomal level of sensory block or unilateral T6 dermatomal level of sensory block, 317 (52.5%), 66 (12.7%), and 68 (16.6%) respondents, respectively, would administer a dose of intrathecal local anesthetic that they use in their routine clinical practice outside the context of a failed epidural top up (Table 4). No significant differences between consultants and trainees were found with respect to this. If the sensory block was unilateral to T6 dermatomal level rather than bilateral to T10 dermatomal level, no significant differences in the dose of intrathecal local anesthetic were found.

### 3.4. Complications of a Neuraxial Technique following a Failed Epidural Top up for a Cesarean Section

Twenty-eight (3.9%) and 250 (35.2%) respondents reported having encountered either a high or total spinal after a CSE and spinal, respectively, in the setting of undertaking these neuraxial techniques after a failed epidural top up of an existing labor epidural for a cesarean section. One-hundred and fifty-seven (22.1%) respondents have encountered an inadequate sensory block requiring general anesthesia following a reduced intrathecal local anesthetic dose for spinal anesthesia after a failed epidural top up for a cesarean section. Eleven (1.5%) respondents have encountered local anesthetic toxicity as a complication of a de novo epidural after a failed epidural top up for a cesarean section.

## 4. Discussion

To our knowledge, this is the first survey to examine clinical decision-making in the context of failed conversion of labor epidural analgesia to cesarean delivery anesthesia. Our survey demonstrated much variation in decision-making consequent to a failed labor epidural top up for a cesarean section. Consistent factors influencing whether or not anesthetic members of the OAA responding to the survey would top up an existing labor epidural for cesarean section were how effective the epidural had been for labor and the urgency of cesarean delivery. Subsequent to a failed epidural top up, factors which influenced management could be attributed to concerns associated with the risks related to the replacement of the epidural, performance of a CSE or spinal, and the induction of general anesthesia. Consensus and guidelines are lacking in this area and may contribute to the variation we found in the clinical management of a failed epidural top up for cesarean section. Complications related to a repeat neuraxial technique in this situation were reported by a significant number of respondents.

If a decision is made to undertake a category-one cesarean section in a parturient with an existing labor epidural, the anesthesiologist is placed in the familiar position of determining whether to convert the epidural analgesia to surgical anesthesia, perform an alternative regional anesthetic technique, or induce general anesthesia. Compared to CSE and spinal anesthesia, general anesthesia has been associated with a shorter decision to delivery interval [7]. Epidural top up has the ability to facilitate a comparable decision to delivery interval to general anesthesia, with a retrospective audit demonstrating a mean decision to delivery interval of 19 and 17 minutes, respectively, for epidural top up and general anesthesia [8]. Relative to bupivacaine or levobupivacaine 0.5% or ropivacaine 0.75%, lidocaine 2% and epinephrine, with or without fentanyl in the epidural top up solution, was associated with the fastest onset of surgical block, leading to a mean difference of 1.7–4.5 minutes in a meta-analysis [9]. The addition of fentanyl at a dose of 50–75 *μ*g further decreased the onset time of surgical block by a mean difference of over 2 minutes. In a recent retrospective cohort study, the operating room to incision interval was shorter for general anesthesia at 6 minutes relative to epidural top up at 11 minutes, but the longer operating room to incision interval did not correlate to inferior neonatal outcomes [7]. Use of general anesthesia, in contrast, has been related to depressed Apgar scores at five minutes, the need for bag mask ventilation, and admission to neonatal intensive care [7, 10].

In the evaluation of whether or not to top up an existing labor epidural for a cesarean section, the most commonly reported factors were a reflection of the underlying evidence. Bauer et al. demonstrated in a meta-analysis that risk factors associated with the failed conversion of labor epidural analgesia were a greater number of unscheduled boluses administered for breakthrough pain in labor, an enhanced urgency for cesarean delivery, and the provision of care by a non-obstetric anesthesiologist [11]. Breakthrough pain in labor could be a marker of a poorly functioning epidural or may signify dysfunctional labor [12]. In a retrospective study, on the other hand, many epidurals which required unscheduled boluses in labor were still found to function well when topped up for a cesarean section [13]. Compared to non-obstetric anesthesiologists, obstetric anesthesiologists could be more experienced in managing problematic labor epidurals and may be more likely to replace poorly functioning epidural catheters before the need for cesarean delivery arises.

It is less clear as to whether the body mass index, weight, cervical dilatation at the time of epidural placement, CSE versus standard epidural technique in labor, and the duration of epidural analgesia increase the likelihood of a failed epidural top up for cesarean section [4, 11]. Obesity has been related to difficult neuraxial block placement, epidural catheter displacement, unfavorable airway examination, and higher rates of cesarean section, all of which could encourage the more careful monitoring and management of epidural analgesia [14, 15]. Use of CSE relative to epidural labor analgesia has been associated with a decreased incidence of overall failure, defined as inadvertent dural puncture, intravenous epidural catheter, inadequate analgesia or no sensory block, or Epidural replacement [16]. Confirmation of the free flow of cerebrospinal fluid during the spinal component of CSE facilitates improved epidural space identification and implies optimal midline placement of the epidural needle, while the resulting small dural hole may act as a conduit to augment the action of local anesthetics injected into the epidural space [17]. Nevertheless, the increased dose of local anesthetic administered in epidural anesthesia for cesarean section could overshadow any effect secondary to the leakage of local anesthetic through the dural hole. The likelihood of epidural catheter migration has been proposed to be greater with an increasing duration of epidural analgesia, but this has not been clinically shown to be the case [13, 14].

Several recommendations have been made in order to decrease the risk of failed labor epidural to surgical anesthesia top up [4]. In the delivery room before any decision to proceed to cesarean section, early recognition of poorly functioning labor epidural analgesia provides the anesthesiologist with an opportunity to manipulate or replace the epidural catheter. If the obstetrician expresses concern about a parturient's slow progress in labor or the fetal heart rate tracing, the anesthesiologist must re-evaluate how well the labor epidural is functioning in anticipation of the need to convert to surgical anesthesia. Should sufficient time be available in the operating theatre after the decision to proceed to cesarean section, the function of the labor epidural can be tested by administering one-quarter to one-third of the full dose of local anesthetic and examining for the level and density of block after approximately five to ten minutes.

If conversion of labor epidural analgesia to cesarean delivery anesthesia fails, deciding upon the most appropriate and safest management step can be difficult. Subsequent options for management can all introduce potential anesthetic risk to the parturient [4, 5]. Decisive factors influencing management, determined in this survey, included the length of time needed to establish a sensory block and the urgency of cesarean section which could, in part, explain the choice of most respondents not to manipulate or replace the epidural in the different scenarios. Further administration of local anesthetic can moreover increase the risk of local anesthetic systemic toxicity [18], a complication encountered by some respondents. If unilateral sensory block occurs, however, the unfavorable location of the epidural catheter, either positioned too lateral in the epidural space or outside the epidural space after passing through the intervertebral foramen, can be corrected by withdrawal. In a retrospective analysis, withdrawal of the epidural catheter followed by the administration of additional surgical anesthetic concentration of local anesthetic was identified as an effective intervention in more than four-fifths of cases of failed epidural top up for cesarean anesthesia [19].

In all the various scenarios of failed epidural conversion, the performance of a spinal was commonly preferred by respondents. It can be a challenge to perform a spinal in this setting because of the associated difficulty in obtaining cerebrospinal fluid, which may be due to collapse of the subarachnoid space below the termination of the spinal cord secondary to the volume effect of the epidural bolus [20, 21]. Spinal anesthesia performed within half an hour of a failed epidural top up has been associated with an increased risk of failure and might reflect the erroneous assumption that the free flow of clear fluid must be cerebrospinal fluid rather than previously injected local anesthetic within the epidural space [22].

Our survey found that if the dermatomal level of inadequate sensory block was higher or unilateral, then a lower dose of intrathecal local anesthetic for spinal anesthesia and alternative options for management were favored by respondents. Such findings may reflect concerns about the risk of high and total spinal block when performing a spinal after a failed epidural top up [23–25], the incidence of which has been reported to be as high as 11% [26]. The increased likelihood of high or total spinal in this context could be secondary to the preexisting subclinical analgesia caused by previous exposure of the neuronal tissue to epidural local anesthetic solution [27], compression of the dural sac by residual local anesthetic in the epidural space resulting in cephalad displacement of the intrathecally injected drugs [20, 28], and the leakage of local anesthetic through the dural hole into the subarachnoid space [27, 29]. Measures recommended to decrease the risk of high and total spinal block include performing the spinal in the sitting position, reducing the dose of intrathecal bupivacaine by 20%, and delaying supine positioning following spinal injection [26, 30]. Decreasing the dose of intrathecal local anesthetic can, however, increase the likelihood of block failure [31], a complication reported by numerous respondents.

Use of a CSE can facilitate a decreased initial dose of intrathecal local anesthetic to be administered with a reduced risk of block failure due to the ability to provide further local anesthetic doses as needed through the epidural catheter [31, 32]. Over a third of respondents have encountered a high or total spinal after a spinal but this complication was reported almost ninefold less subsequent to a CSE. Concerns about the risk of the untested epidural catheter were present amongst some respondents, despite evidence that the occurrence of a failed epidural component is unlikely after a successful CSE [33]. Individual studies report longer performance times for CSE compared to spinal [34], but only one trial showed a clinically meaningful difference of 11 minutes [35]. General anesthesia has been associated with accidental awareness and complications related to aspiration and failed intubation, with critical incidents mainly occurring after conversion of regional anesthesia rather than primary general anesthesia [36, 37].

It is the opinion of the authors that if the level and density of objective block does not increase once sufficient time has passed after attempted conversion of labor epidural analgesia to surgical anesthesia, then a CSE with a decreased initial dose of intrathecal local anesthetic to minimize the risk of a high or total spinal is the preferred technique should the fetal condition allow.

Our survey had a number of potential limitations. The scenarios describing failed epidural top up for cesarean delivery could have been subject to differences in interpretation by individual respondents. It is likely that not all factors which influenced management were considered, and this included the presence of local and regional protocols or working in a district general rather than a teaching hospital. The response rate of 41% was lower than expected but was reasonable relative to previously published surveys [38, 39], although a higher response rate would have been preferable [40]. Surveying members of OAA, however, would have resulted in a selection bias towards a subgroup of anesthetic providers with an interest in obstetric anesthesia and the reported practice was more likely to represent evidence based and optimal management.

## 5. Conclusions

In conclusion, the survey provides insight into factors influencing whether or not anesthesiologists with an obstetric interest would convert an existing labor epidural for cesarean delivery and reveals variability in decision-making following a failed epidural top up for cesarean section. In uncovering the most common clinical practice preferences amongst anesthesiologists in this setting, the results from this survey can support the development of best practice guidelines.

## Figures and Tables

**Table 1:** Factors which influence whether or not respondents top up an existing labor epidural for a cesarean section.

Influencing factor	Respondents (*n*=710)
How effective the epidural has been for labor pain	701 (98.7)
Category of cesarean section	519 (73.1)
Dermatomal level of blockade	434 (61.1)
Current pain score with contractions	355 (50.0)
Assessment of airway	266 (37.5)
Maternal preference	254 (35.8)
Body mass index	180 (25.4)
Length of time epidural has been in situ	106 (14.9)
Fasting status	92 (13.0)
Other	42 (5.9)
Labor neuraxial technique (CSE or epidural)	37 (5.2)
Age	2 (0.3)

Data are presented as number (%). CSE = combined spinal-epidural.

**Table 2:** Factors which influence management after a failed epidural top up of an existing labor epidural for a cesarean section.

Influencing factor	Respondents (*n*=710)
Category of cesarean section	650 (91.5)
Dermatomal level of blockade	554 (78.0)
Assessment of airway	551 (77.6)
Risk of high or total spinal	513 (72.3)
Pattern of neuraxial block failure such as unequal or unilateral block	484 (68.2)
Perceived potential difficulty in achieving a neuraxial block	449 (63.2)
Length of time needed to establish a sensory block	433 (61.0)
Body mass index	431 (60.7)
Difficulties in predicting the correct intrathecal local anesthetic dose needed	410 (57.7)
Concentration and volume of local anesthetic used in epidural top up	386 (54.4)
Fasting status	237 (33.4)
Extension of sensory block possible if needed with a CSE or epidural technique	199 (28.0)
Risk of local anesthetic toxicity	179 (25.2)
Risk of the untested epidural catheter with a CSE technique	87 (12.3)
Postoperative analgesia	78 (11.0)
Other	29 (4.1)

Data are presented as number (%). CSE = combined spinal-epidural.

**Table 3:** Usual next management step of respondents if a top up of an existing labor epidural for a category-two cesarean section resulted in an inadequate or failed sensory block^*∗*^.

Management	No objective sensory block (*n*=709)	Bilateral T10 sensory block (*n*=699)	Unilateral T6 sensory block (*n*=691)	*p* value (no objective sensory block vs bilateral T10 sensory block)	*p* value (bilateral T10 sensory block vs unilateral T6 sensory block)
CSE	87 (12.3)	129 (18.5)	105 (15.2)	<0.001	0.10
General anesthesia	67 (9.4)	120 (17.2)	150 (21.7)	<0.001	0.03
Repeat epidural	2 (0.3)	11 (1.6)	13 (1.9)	0.01	0.66
Spinal	524 (73.9)	398 (56.9)	310 (44.9)	<0.001	<0.001
Withdraw in situ epidural catheter	6 (0.8)	10 (1.4)	65 (9.4)	0.30	<0.001
Other	23 (3.2)	31 (4.4)	48 (6.9)	0.25	0.04

Data are presented as number (%). CSE = combined spinal-epidural. ^*∗*^In these scenarios, respondents were told to assume that neither further epidural top ups nor time would result in any change in the dermatomal level of the sensory block, and assessment of the parturient would demonstrate no undue concerns about the airway and no obvious difficulties in achieving a neuraxial technique if needed.

**Table 4:** Dose of intrathecal local anesthetic which would be used, compared to that used in their routine clinical practice, by respondents who selected to perform a combined spinal-epidural or spinal as their usual next management step after a top up of an existing labor epidural had resulted in an inadequate or failed sensory block for a category-two cesarean section.

Dose of intrathecal local anesthetic	No objective sensory block (*n*=604)	Bilateral T10 sensory block (*n*=520)	Unilateral T6 sensory block (*n*=409)	*p* value (no objective sensory block vs bilateral T10 sensory block)	*p* value (no objective sensory block vs unilateral T6 sensory block)
Normal	317 (52.5)	66 (12.7)	68 (16.6)	<0.001	<0.001
75 to <100% of normal	206 (34.1)	188 (36.2)	134 (32.8)	0.39	0.75
50 to <75% of normal	70 (11.6)	213 (41.0)	150 (36.7)	<0.001	<0.001
25 to <50% of normal	3 (0.5)	43 (8.3)	45 (11.0)	<0.001	<0.001
<25% of normal	0 (0)	2 (0.4)	6 (1.5)	0.13	0.003
Other	8 (1.3)	8 (1.5)	6 (1.5)	0.75	0.83

Data are presented as number (%).

## Data Availability

The data used to support the findings of this study are available from the corresponding author upon request.

## Supplementary Materials

Supplementary Materials Content and structure of obstetric survey.
