# Supplementary material for: Labor Epidural Analgesia to Cesarean Section Anesthetic Conversion Failure: A National Survey
Source: Anesthesiol Res Pract. 2019 Jun 2;2019:6381792. doi: 10.1155/2019/6381792 (PMC6589285; doi:10.1155/2019/6381792)
Supplement: Supplementary Materials — Content and structure of obstetric survey. [file 6381792.f1.pdf]

Q1. Grade of Anaesthetist (Tick one)

Consultant
Associate Specialist
Staff Grade
ST5-7
ST3-4
SHO

Q2. In your clinical practice, what is your standard anaesthetic technique for a category four caesarean section? (Tick one)

Combined spinal-epidural
Epidural
General anaesthesia
Spinal

Q3. When evaluating as to whether or not to top up an existing labour epidural for a caesarean section, which factors, if any, would influence your decision? (Tick all that apply)

Maternal age
Maternal body mass index
Airway assessment
Fasting status
Maternal preference
Labour epidural insertion technique: combined spinal-epidural or epidural
Length of time epidural has been in situ
How effective the epidural has been thus far in the management of labour pain
Current pain score with contractions
Assessment of sensory level of block prior to top ups
Category of caesarean section
Other

Q4. Would you consider topping up an existing labour epidural for a category one caesarean section? (Tick one)

Yes
No

Q5. Consider a scenario where an epidural top up of an existing labour epidural has resulted in no objective sensory block for a category two caesarean section. Assume further epidural top ups or time will still result in no detectable block with the epidural catheter in the position it is in. Assessment of the patient demonstrates no undue concerns about the airway and no obvious potential difficulties in achieving a neuraxial technique if needed. In such a case, what would your usual next management step be? (Tick one)

Combined spinal-epidural
General anaesthesia *(Go next to Q7)*
Repeat epidural *(Go next to Q7)*
Spinal
Withdraw epidural catheter already in situ *(Go next to Q7)*
Other *(Go next to Q7)*

Q6. Given this scenario with no objective sensory block, what dose of intrathecal local anaesthetic, compared to what you use in your routine clinical practice, would you use for this particular patient? (Tick one)

Normal spinal local anaesthetic dose
75 to <100% of normal spinal local anaesthetic dose
50 to <75% of normal spinal local anaesthetic dose
25 to <50% of normal spinal local anaesthetic dose
<25% of normal spinal local anaesthetic dose
Other

Q7. Consider a scenario where an epidural top up of an existing labour epidural has resulted in a good BILATERAL T10 sensory block to temperature change for a category two caesarean section. Assume further epidural top ups or time will still result in no detectable block with the epidural catheter in the position it is in. Assessment of the patient demonstrates no undue concerns about the airway and no obvious potential difficulties in achieving a neuraxial technique if needed. In such a case, what would your usual next management step be? (Tick one)

Combined spinal-epidural
General anaesthesia *(Go next to Q9)*
Repeat epidural *(Go next to Q9)*
Spinal
Withdraw epidural catheter already in situ *(Go next to Q9)*
Other *(Go next to Q9)*

Q8. Given this scenario where the epidural top up has resulted in a good BILATERAL T10 sensory block to temperature change, what dose of intrathecal local anaesthetic, compared to what you use in your routine clinical practice, would you use for this particular patient? (Tick one)

Normal spinal local anaesthetic dose
75 to <100% of normal spinal local anaesthetic dose
50 to <75% of normal spinal local anaesthetic dose
25 to <50% of normal spinal local anaesthetic dose
<25% of normal spinal local anaesthetic dose
Other

Q9. Consider a scenario where an epidural top up of an existing labour epidural has resulted in a good UNILATERAL T6 sensory block to temperature change for a category two caesarean section. Assume further epidural top ups or time will still result in no detectable block with the epidural catheter in the position it is in. Assessment of the patient demonstrates no undue concerns about the airway and no obvious potential difficulties in achieving a neuraxial technique if needed. In such a case, what would your usual next management step be? (Tick one)

Combined spinal-epidural
General anaesthesia *(Go next to Q11)*
Repeat epidural *(Go next to Q11)*
Spinal
Withdraw epidural catheter already in situ *(Go next to Q11)*
Other *(Go next to Q11)*

Q10. Given this scenario where the epidural top up has resulted in a good UNILATERAL T6 sensory block to temperature change, what dose of intrathecal local anaesthetic, compared to what you use in your routine clinical practice, would you use for this particular patient? (Tick one)

Normal spinal local anaesthetic dose
75 to <100% of normal spinal local anaesthetic dose
50 to <75% of normal spinal local anaesthetic dose
25 to <50% of normal spinal local anaesthetic dose
<25% of normal spinal local anaesthetic dose
Other

Q11. In making these decisions about what to do next after a failed epidural top up for caesarean section, which of the following factors, if any, would influence what you did? (Tick all that apply)

Assessment of maternal airway
Body mass index
Fasting status
Concentration and volume of local anaesthetic used in epidural top up
Dermatomal level of the block
Pattern of neuraxial block failure such as unequal or unilateral block
Length of time needed to establish a sensory block
Perceived potential difficulty in achieving a neuraxial block
Extension of sensory block possible if needed with a combined spinal-epidural or epidural technique
Risk of the untested epidural catheter with a combined spinal-epidural technique

Difficulties in predicting the correct dose of intrathecal local anaesthetic needed

Maternal postoperative analgesia

Risk of high or total spinal

Risk of local anaesthetic toxicity

Category of caesarean section

Other

Q12. Have you ever encountered a high or total spinal as a complication of a combined spinal-epidural or spinal after a failed epidural top up for caesarean section? (Tick all that apply)

Yes, as a complication of a combined spinal-epidural after a failed epidural top up

Yes, as a complication of a spinal after a failed epidural top up

No

Q13. Have you ever encountered an inadequate sensory block requiring general anaesthesia following a reduced spinal dose after a failed epidural top up for caesarean section? (Tick one)

Yes

No

Q14. Have you ever encountered local anaesthetic toxicity as a complication of a de novo epidural top up after a failed epidural top up for caesarean section? (Tick one)

Yes

No
